# Optimal Surgical Strategy for Kommerell’s Diverticulum Associated with a Right-Sided Aortic Arch: A Report of Four Cases

**DOI:** 10.70352/scrj.cr.25-0049

**Published:** 2025-06-18

**Authors:** Yumeka Tamai, Tatsuya Ogawa, Ryusuke Hamada, Genichi Sakaguchi

**Affiliations:** Department of Cardiovascular Surgery, Kindai University Faculty of Medicine, Osakasayama, Osaka, Japan

**Keywords:** right-sided aortic arch, Kommerell’s diverticulum, aberrant subclavian artery, cardiovascular surgery

## Abstract

**INTRODUCTION:**

Kommerell’s diverticulum is often associated with a right-sided aortic arch. It presents as a saccular aneurysm. Although various surgical strategies have been reported, optimal treatment has not been established.

**CASE PRESENTATION:**

Four patients with right-sided aortic arch underwent different surgeries for Kommerell’s diverticulum. The pattern of aortic arch was a mirror-image of the normal left aortic arch in Cases 1 and 2. In Cases 3 and 4, it was right-sided aortic arch with an aberrant left subclavian artery as its last branch. Cases 1 and 3 presented with compression symptoms caused by Kommerell’s diverticulum. They underwent open surgery or thoracic endovascular aortic repair through the different approaches. Their postoperative courses were favorable.

**CONCLUSIONS:**

The surgical strategy for Kommerell’s diverticulum with a right-sided aortic arch should be selected based on the anatomical characteristics of the cervical vessels, compression symptoms, and surgical risks.

## Abbreviations

ALSCA
: aberrant left subclavian artery
DAR
: descending aorta replacement
FET
: frozen elephant trunk
KD
: Kommerell’s diverticulum
RAA
: right-sided aortic arch
TAR
: total aortic arch replacement
TEVAR
: thoracic endovascular aortic repair

## INTRODUCTION

KD associated with a RAA lacks an established treatment protocol. We experienced 4 patient-specific surgical techniques, tailored to anatomical features, compression symptoms, and frailty level.

## CASE PRESENTATION

### Case 1

**Fig. 1:**
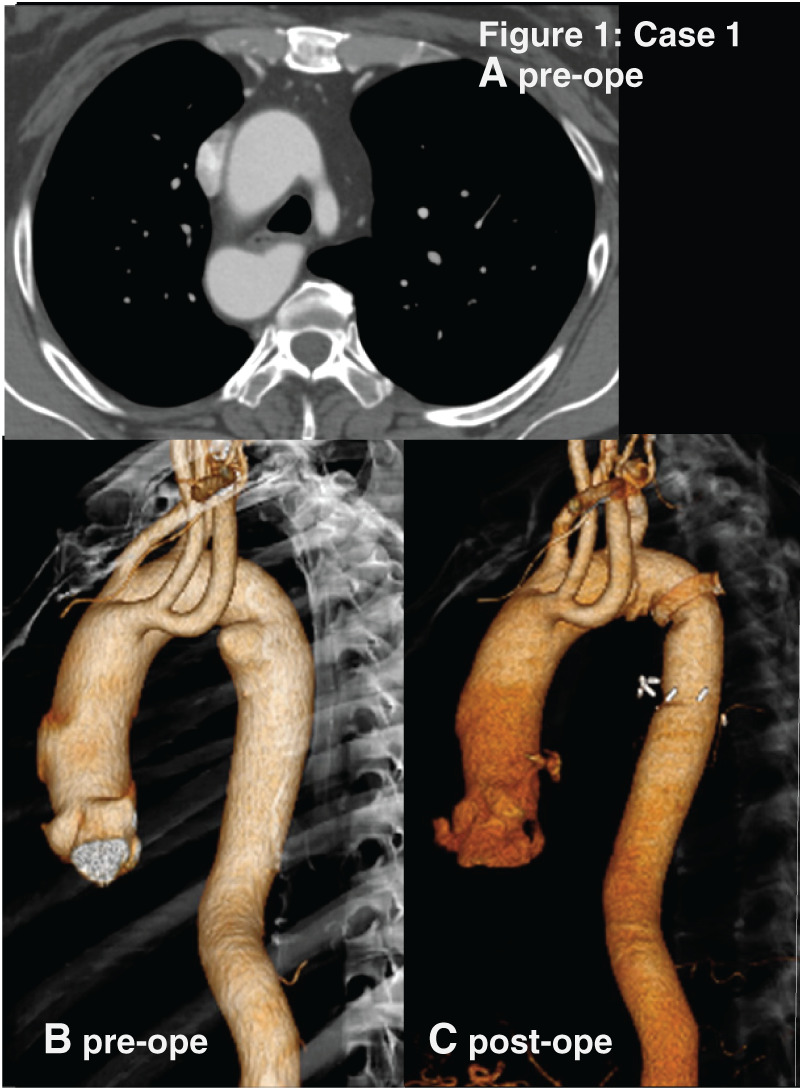
The figure 1 correspond to the images of Case 1 respectively. (**A**) Preoperative enhanced CT images, axial view. (**B**) Preoperative 3D-reconstructide CT. (**C**) Postoperative 3D-reconstructed CT. Case 1 are mirror-image RAAs. RAA, right-sided aortic arch

A 49-year-old woman with dysphagia for several years. CT revealed RAA and KD on the distal arch protruding by 19 mm. Her esophagus was compressed dorsally by KD. The pattern of RAA was a mirror-image of the normal left aortic arch. Aortic arch and descending aorta ran on the right side of the spine (**Fig. 1**).

Descending aorta replacement was performed through right thoracotomy.

The proximal clamp was placed between the right common carotid artery and the right subclavian artery. This was to avoid interference with the left recurrent nerve in the distal arch, the azygos vein crossing the proximal thoracic descending aorta, and the vulnerable tissue of the KD origin. Also, distal thoracic aorta and right subclavian artery were clamped during anastomosis.

Dysphagia improved within several months after the operation.

### Case 2

**Fig. 2:**
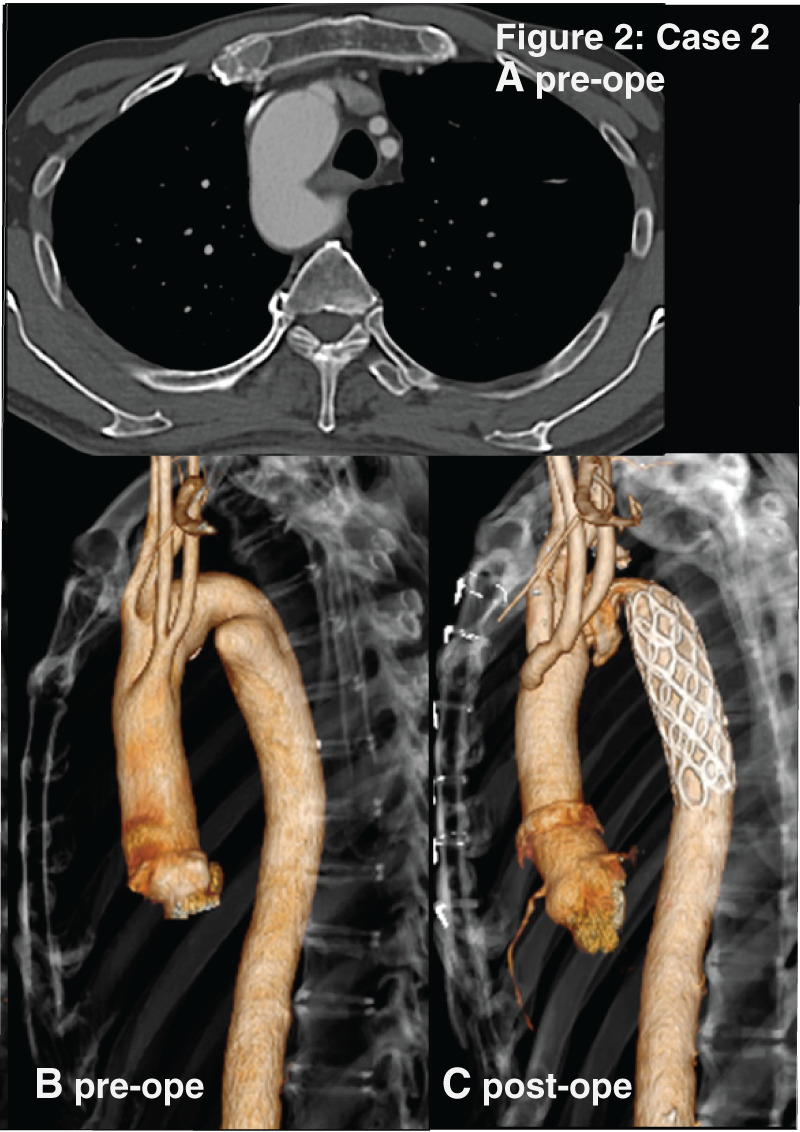
The figure 2 correspond to the images of Case 2 respectively. (**A**) Preoperative enhanced CT images, axial view. (**B**) Preoperative 3D-reconstructide CT. (**C**) Postoperative 3D-reconstructed CT. Case 2 are mirror-image RAAs. RAA, right-sided aortic arch

A 70-year-old asymptomatic man, current smoker. CT showed mirror-image RAA and KD on the descending thoracic aorta with the sac depth of 14 mm (**Fig. 2**).

Surgical intervention was indicated for KD due to its high risk of rupture as a saccular aneurysm based on high sac/neck ratio (**Table 1**). Total aortic arch replacement and insertion of FET were performed through median sternotomy. The extremely steep angle of 30 degrees in the aortic arch raised concerns about procedural difficulty and potential vascular injury, TEVAR was not selected. Graft replacement with thoracotomy was also avoided to prevent respiratory complications, because of the patient’s current smoking status and a significant history of heavy smoking. Postoperative CT showed successful exclusion of the diverticulum. While CT revealed a kink in the FET at the apex of the aortic arch, the ankle–brachial index did not decrease.

**Table 1:** Patients’ characteristics and treatment courses

Case	Age	ALSCA	Symptom	Operation	Approach	Follow	Horizontal diameter		Vertical diameter		Sac depth/neck width	Vertical/horizontal diameter	Arch angle
							Pre	Post	Pre	Post			
1	49	–	+	DAR	Right thoracotomy	1.3 y	42	22	21	0	19/21 = 0.9	21/42 = 0.5	49
2	70	–	–	TAR + FET	Median sternotomy	1 mon	40	36	20	16	14/16 = 0.9	16/36 = 0.4	33
3	68	+	+	DAR + ALSCA reconstruction	Left thoracotomy	6.5 y	56	27	24	0	24/24 = 1.3	24/56 = 0.4	42
4	72	+	–	TEVAR	Femoral artery	9.0 y	44	31	18	16	23/18 = 1.0	18/44 = 0.4	58

Focus on ALSCA presence and compression symptoms. ALSCA were presented in Cases 3 and 4. Compression symptoms were positive in Cases 1 and 3. Postoperative CT images were obtained during follow-up approximately 1 month after surgery.

ALSCA, aberrant left subclavian artery; DAR, descending aorta replacement; FET, frozen elephant trunk; TAR, total aortic arch replacement; TEVAR thoracic endovascular aortic repair

### Case 3

**Fig. 3:**
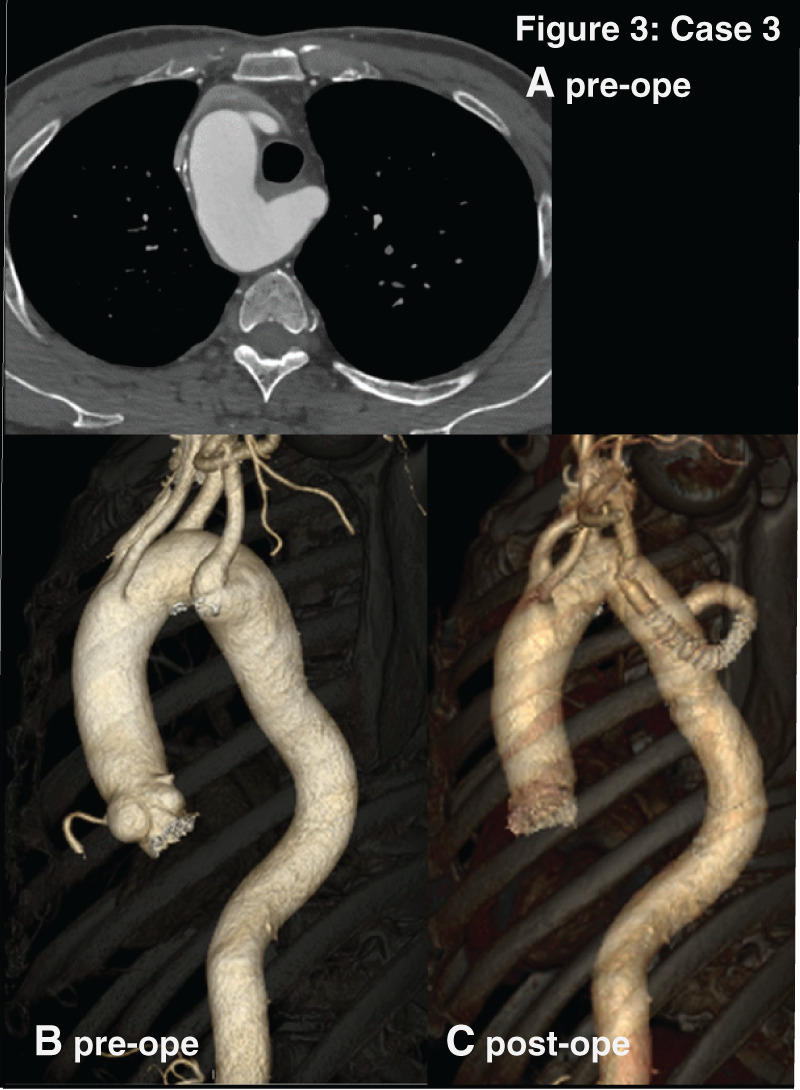
The figure 3 correspond to the images of Case 3 respectively. (**A**) Preoperative enhanced CT images, axial view. (**B**) Preoperative 3D-reconstructide CT. (**C**) Postoperative 3D-reconstructed CT. Case 3 are mirror-image RAAs with ALSCA. ALSCA, aberrant left subclavian artery; RAA, right-sided aortic arch

This is the case of a 68-year-old man with hoarseness and dysphagia for 6 years. CT revealed RAA with an ALSCA as its last branch and KD compressing his esophagus (**Fig. 3**). Descending aorta replacement and ALSCA reconstruction were performed. Due to the accessibility for ALSCA, the left thoracotomy approach was selected. During anastomosis, bleeding from the proximal clamp site near KD necessitated an open proximal method. First, in order to control bleeding, proximal and distal aorta were clamped. The right femoral artery and vein were used for perfusion and drainage initially, but additional lines were established, including ascending aortic perfusion cannula, right atrial drainage cannula, and left ventricular vent from the right upper pulmonary vein. The patient was cooled to 18°C under deep hypothermia, and during anastomosis, low-flow perfusion was maintained via femoral artery. A head-down position and carbon dioxide insufflation to the surgical field was applied.

The postoperative course was uneventful, and the patient’s dysphagia improved during follow-up.

### Case 4

**Fig. 4:**
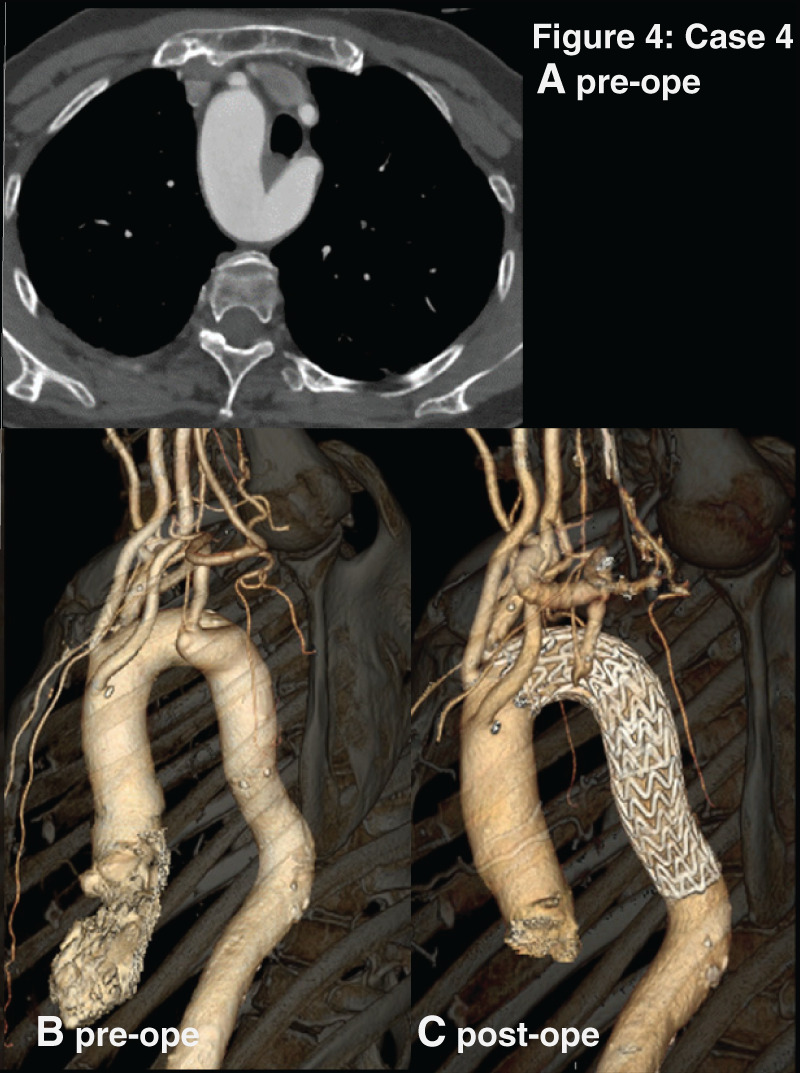
The figure 4 correspond to the images of Case 4 respectively. (**A**) Preoperative enhanced CT images, axial view. (**B**) Preoperative 3D-reconstructide CT. (**C**) Postoperative 3D-reconstructed CT. Case 4 are mirror-image RAAs with ALSCA. ALSCA, aberrant left subclavian artery; RAA, right-sided aortic arch

This is the case of a 72-year-old woman with cognitive decline and frailty. CT revealed the same type of RAA as Case 3. KD with a protrusion diameter of 23 mm was identified at the origin of ALSA (**Fig. 4**).

This patient, similar to Case 2, had a steep aortic arch. Although TAR + FET was considered, an endovascular approach was chosen due to significant frailty and being asymptomatic. Initially, subclavian artery (SCA) embolization was planned. However, intraoperative angiography revealed that the vertebral artery was in close proximity, therefore, embolization was not performed.

One-debranch TEVAR was performed with a left common carotid to ALSCA bypass. Embolization of the ALSCA was not performed because intraoperative angiography revealed that the vertebral artery was so close that there was a risk to be obstructed.

Since the arterial wall surrounding KD is fragile, careful attention was paid to avoid inadvertent wire advancement into the diverticulum during stiff wire manipulation. Additionally, balloon touch-up was cautiously performed to prevent excessive pressure.

Postoperative CT showed no endoleaks or vascular injuries.

In all the four cases, no aorta-related events occurred during follow-up. On the CT scan performed 1 month after surgery, the horizontal diameters decreased in Cases 2 and 4 (**Table 1**).

## DISCUSSION

RAA has 2 primary types: mirror-image of the normal left aortic arch (59%) and RAA with ALSCA (39.5%).^1)^ KD appears in 20%–60% of patients with aberrant subclavian arteries, with 95% being asymptomatic.^2)^

In asymptomatic cases, we used the following criteria to indicate high-risk saccular aneurysms:

- Sac depth/neck width >0.8
- Aspect ratio (vertical diameter/horizontal diameter) <1.0.^3)^

We systematically selected surgical strategies based on the presence of ALSCA, compression symptoms, patient frailty, and comorbidities.

**Fig. 5:**
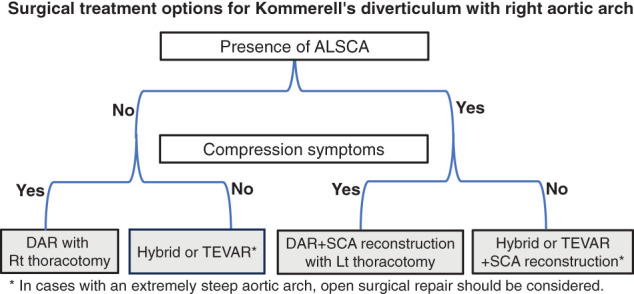
A flowchart outlining our treatment strategy for Kommerell’s diverticulum with right aortic arch. ALSCA, aberrant left subclavian artery; DAR, descending aorta replacement; Rt, right; SCA, subclavian artery; TEVAR, thoracic endovascular aortic repair

Our primary strategy is open surgical replacement of the descending aorta. The approaches—whether via median sternotomy, right thoracotomy, or left thoracotomy—are selected according to the morphology of the RAA, particularly based on the presence or absence of ALSCA. In the absence of ALSCA, descending aorta replacement with right thoracotomy is considered. In cases with ALSCA, we prefer left thoracotomy with graft replacement and in situ reconstruction of the right subclavian artery. However, in asymptomatic cases, hybrid procedures or TEVAR may be considered depending on patient frailty and comorbidities. Additionally, if the aortic arch is excessively steep, the appropriateness of endovascular treatment should be carefully reconsidered (**Fig. 5**). However, the specific angulation of the aortic arch that constitutes a procedural risk remains unclear.

Open surgical repair is traditionally performed through lateral thoracotomy to allow access to ALSCA reconstruction and manage fragile tissue of KD origins. However, descending replacement with thoracotomy is basically highly invasive, and carries risks such as respiratory complications and chylothorax.^2,4)^

ALSCA management options include carotid-subclavian bypass, subclavian-carotid transposition, in situ reconstruction, or simple ligation.^5)^

While reports indicate no ischemic symptoms after ALSCA ligation, the risk of spinal cord ischemia remains uncertain.^4)^ Since bypass reconstruction does not significantly increase surgical invasiveness, we opted for bypass as the preferred strategy. Regardless of the technique, the KD origin remains fragile, requiring careful handling to prevent injury.

Hybrid approaches such as FET or TEVAR are potential options for asymptomatic cases. In recent years, TEVAR has been applied for KD with ALSCA in 12%.^2)^ However, endoleaks—especially type 2 from the subclavian artery and type 1 due to inadequate proximal landing—are concerns. Reports indicate that type 1 endoleaks occur in 5.9%–17.9% of cases, while type 2 endoleaks occur in 7.6%–14.3%.^5–7)^ Retrograde type A aortic dissection is another risk, reported in approximately 1.5% of cases. Reintervention rates vary from 5.1% to 5.9%, with a small percentage requiring late conversion to open surgery.^5–7)^

In cases with steep RAA morphology, excessive kinking or endoleaks may be problematic with TEVAR. In Case 2, the aortic arch angle was particularly steep, raising concerns that TEVAR or FET might have been suboptimal choices despite the lack of postoperative complications. While performing a distal anastomosis beyond the inflection point of the arch could provide a solution, it would likely be technically challenging through a median sternotomy. Because intraoperative evaluation of the extension and expansion of the stentgraft is not conducted with the FET technique, TEVAR might have been more suitable, as it allows intraoperative assessment of potential kinking or injury and provides an option for additional procedures such as balloon dilation. When performing TEVAR, given the fragility of KD tissue, meticulous wire manipulation is crucial to avoid wire misplacement into the diverticulum and excessive balloon pressure. For stentgraft sizing, excessive oversizing was avoided. Additionally, since right-sided aortas frequently exhibit tortuosity even in the descending thoracic segment, the distal landing zone was set to ensure placement within a straight portion of the vessel.

Recent reports have shown that KD can regress after hybrid or endovascular procedures,^6,8)^ and similar outcomes were observed in the present 2 cases. These findings suggest that even in symptomatic cases, hybrid or TEVAR may be feasible for frail patients.

One systematic review reported that overall 30-day mortality rates for open surgery, hybrid approaches, and TEVAR were reported as 3.5%, 6.8%, and 3.9%, respectively, with no significant differences.^7)^ These outcomes are comparable to those reported for descending aortic replacement performed for indications other than KD, reported as mortality rates of approximately 3.5%–11% for open surgery and 2%–3% for TEVAR.^9,10)^ However, given the diverse pathophysiology, it is essential to continually evaluate and select the optimal surgical strategy to further improve prognosis.

## CONCLUSIONS

Preoperative assessment of KD associated with RAA should include ALSCA presence, presence of symptoms, frailty level, and aortic arch sharpness. These factors guide a systematic selection of surgical strategies. However, given the variation in KD pathology and advancements in surgical techniques, individualized case evaluation remains essential.

## DECLARATIONS

### Funding

None.

### Authors’ contributions

All of the authors contributed to the treatment of the patients.

YT contributed to the drafting of the manuscript.

TO and RH edited the manuscript.

TO and GS supervised.

All the authors read and approved the final manuscript.

### Availability of data and materials

Not applicable.

### Ethics approval and consent to participate

This work does not require ethical considerations or approval. Informed consent to participate in this study was obtained from the patient.

### Consent for publication

All the patients provided informed consent for the publication of this case report.

### Conflict of interest

None declared.
